# Stability and hydrogen storage performance of Na_2_LiXH_6_ (X = Zr, V, Cr) double perovskite hydrides *via* DFT and AIMD

**DOI:** 10.1039/d5ra08708b

**Published:** 2026-01-06

**Authors:** Muhammad Kaleem, Malik Muhammad Asif Iqbal, Asif Nawaz Khan

**Affiliations:** a Material Research Laboratory (MRL), Department of Physics, International Islamic University H-10 Islamabad 44000 Pakistan; b Department of Chemistry, University of Okara 56300 Pakistan mmasif101@gmail.com; c Materials Modeling and Simulation Lab, Department of Physics, University of Science & Technology Bannu 28100 Khyber Pakhtunkhwa Pakistan

## Abstract

This study aims to provide a comprehensive first-principles investigation, based on density functional theory (DFT) using the GGA-PBE functional, of Na_2_LiXH_6_ (X = Zr, V, Cr) double perovskite hydrides that crystallize in the *Fm*3̄*m* (225) space group. The structural, electronic, optical, and thermodynamic properties were systematically explored to evaluate their potential for advanced hydrogen storage and clean energy applications. Phonon dispersion and *ab initio* molecular dynamics (AIMD) simulations confirm the dynamical and thermal stability of the system at 300 K, without any structural distortion. Among the investigated compounds, Na_2_LiVH_6_ shows the highest gravimetric capacity (5.50 wt%) and an optimal desorption temperature (540.23 K), favorable for reversible hydrogen release. Electronic analysis reveals metallic conductivity, while thermodynamic parameters, including heat capacity, enthalpy, entropy, and free energy, exhibit stable temperature-dependent trends. Collectively, these DFT-GGA-PBE results demonstrate that Na_2_LiVH_6_ possesses superior mechanical endurance, lattice stability, and multifunctional potential for next-generation hydrogen storage and sustainable energy applications.

## Abstract 2

This study aims to provide a comprehensive first-principles investigation, based on density functional theory (DFT) using the GGA-PBE functional, of Na_2_LiXH_6_ (X = Zr, V, Cr) double perovskite hydrides that crystallize in the *Fm*3̄*m* (225) space group.

## Graphical abstract

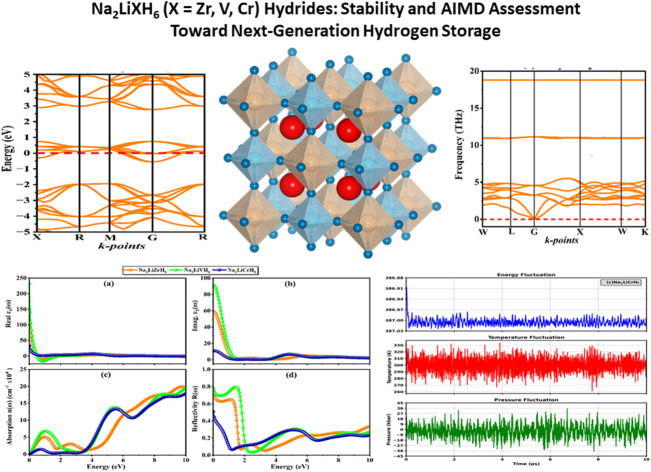

## Introduction

The increasing global energy needs and escalating environmental degradation necessitate the rapid advancement of sustainable energy technologies. Among the different alternative fuels, hydrogen has emerged as a highly promising energy carrier due to its exceptional energy density and clean combustion products.^1^ Nevertheless, its large-scale utilization is still limited by difficulties associated with safe, efficient, and practical storage and transportation.^2^ Traditional storage techniques, such as high-pressure gas compression, cryogenic liquid storage, and cryo-compressed systems, demand significant energy to maintain extreme pressure or low-temperature conditions and are further challenged by unavoidable boil-off losses.^3,4^ Alternatively, solid-state hydrogen storage has emerged as a focal area of research owing to its superior volumetric density, enhanced safety, and reversible storage capability.^5,6^ A wide range of solid materials has been investigated, including intermetallic alloys,^7,8^ metal hydrides,^9–12^ graphene-based nanostructures,^13^ metal–organic frameworks (MOFs),^14,15^ MXenes,^16,17^ and liquid organic hydrogen carriers (LOHCs).^18^ Among these, metal hydrides are particularly attractive because hydrogen is chemically bonded under moderate conditions, offering high volumetric capacities and intrinsic safety. Furthermore, their storage performance can be optimized through compositional engineering and catalytic modification.^19,20^

Perovskite hydrides have recently emerged as a promising subclass of ternary hydrides with remarkable promise for hydrogen storage applications. These materials possess the ability to absorb and retain hydrogen both on their surfaces and within their crystal frameworks, offering an efficient approach to overcoming the persistent challenges of hydrogen storage and transport.^21^ Their high gravimetric capacity, excellent thermal and cyclic stability, and favorable reversibility make them highly attractive for next-generation solid-state hydrogen systems. Various perovskite hydrides such as NaMgH_3_, KAlH_3_, and NaAlH_3_ have been synthesized and characterized, exhibiting orthorhombic or cubic crystal symmetries with remarkable thermodynamic stability.^22,23^ Reported hydrogen storage capacities for AVH_3_ (A = Na, K, Rb, Cs), XPtH_3_ (X = Li, Na, K, Rb), and XFeH_3_ (X = Ca, Sr, Ba) fall within 1–5 wt%, highlighting their competitive storage performance.^24–26^ Double perovskite systems, including Na_2_LiAlH_6_ and K_2_LiAlH_6_, show dehydrogenation capacities of approximately 3.09 wt% and 5.2 wt%, respectively.^27–29^ Li-doped K_2_NaAlH_6_ demonstrates an improved storage capacity of 4.91 wt% with increasing Li concentration,^30^ while substitution of alkaline earth metals in A_2_KNaH_6_ (A = Be, Mg, Ca) further enhances capacity to 8.57, 5.19, and 4.1 wt%, respectively.^31^ Moreover, K_2_XAlH_6_ (X = Li, Na) hydrides exhibit wide band gaps and strong mechanical stability under external stress.^32^ Despite these advances, many double perovskite hydrides remain insufficiently examined, underscoring the need for detailed AIMD investigations to optimize their hydrogen storage characteristics and practical applicability.

In this study, Na_2_LiXH_6_ (X = Zr, V, Cr) double perovskite hydrides are investigated using DFT to explore their fundamental structural, electronic, mechanical, optical, and thermodynamic behaviors. The study aims to identify their feasibility and effectiveness as potential prospects for future solid-state H_2_ storage technologies. Despite the growing interest in complex hydrides, these specific Zr, V, and Cr based Na_2_LiXH_6_ (X = Zr, V, Cr) compounds have not yet been systematically explored, either computationally or experimentally. The strategic substitution of X-site cations with varying electronegativities and ionic radii introduces tunable effects on formation energy, stability, electronic structure, and hydrogen release thermodynamics, an aspect that remains largely unreported in existing literature. To bridge this gap, DFT calculations were conducted to elucidate the atomic-level bonding interactions, energetic characteristics, and hydrogen storage potential of the compounds. Complementary AIMD simulations were employed to evaluate their thermal robustness. The results reveal the structural and energetic stability of these previously unexplored perovskite hydrides, providing a theoretical basis for the rational design of advanced materials for H_2_ storage and energy conversion applications.

## Computational methodology

All computational analyses were conducted using DFT within the CASTEP simulation package^33^ to investigate the ground-state characteristics and optimized structural configurations of Na_2_LiXH_6_ (X = Zr, V, Cr) double perovskite hydrides. The Kohn–Sham equations were implemented with a plane-wave basis set, applying a kinetic energy cutoff of 600 eV,^34^ and ultrasoft pseudopotentials were utilized to model the interactions between electrons and ions accurately. The exchange correlation interactions were described within the framework of the generalized gradient approximation (GGA) using the Perdew–Burke–Ernzerhof (PBE) functional.^35^ Structural optimization was performed through the Broyden–Fletcher–Goldfarb–Shanno (BFGS) minimization algorithm, applying strict convergence thresholds of 2 × 10^−5^ eV per atom for total energy, 0.05 eV Å^−1^ for atomic forces, 0.1 GPa for stress, and 0.002 Å for atomic displacements.^36^ For Brillouin zone integration, a Monkhorst–Pack *k*-point mesh of 6 × 6 × 6 was utilized to ensure accurate sampling. After full relaxation, all Na_2_LiXH_6_ (X = Zr, V, Cr) double perovskite hydrides were verified to adopt a cubic symmetry within the *Fm*3̄*m* (No. 225) space group, characterized by lattice angles *α* = *β* = *γ* = 90°, thereby confirming their crystallographic and structural stability.^37^ To evaluate the thermal stability, AIMD simulations were carried out using the pw.x module of Quantum ESPRESSO within the canonical (NVT) ensemble. The simulations employed the velocity Verlet integration algorithm coupled with a Berendsen thermostat fixed at 300 K to maintain temperature control. A time step of 0.96756 fs was adopted for 10 000 iterations, corresponding to a total simulation duration of approximately 9.7 ps. The calculations utilized ultrasoft pseudopotentials in combination with the PBEsol exchange-correlation functional, while the plane-wave and charge density cutoff energies were set to 60 Ry and 400 Ry, respectively. Convergence criteria were defined as 1.0 × 10^−8^ Ry for self-consistent field (SCF) cycles, 1.0 × 10^−5^ Ry for total energy, and 1.0 × 10^−4^ Ry bohr^−1^ for forces, ensuring high numerical precision throughout the simulations.^38^ These computational settings ensured robust convergence and high numerical precision across all investigated systems.

## Results and discussion

### Structural stability

**Fig. 1:**
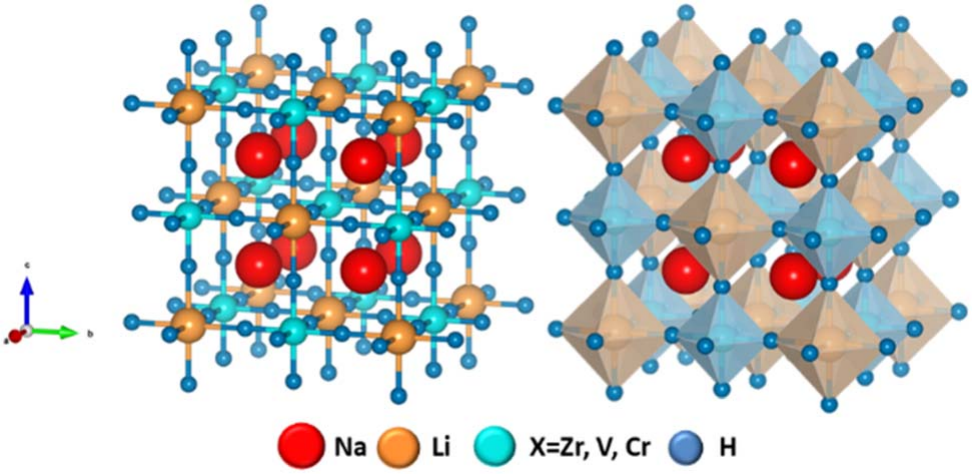
Optimized crystal structure of Na_2_LiXH_6_ (X = Zr, V, Cr) perovskite hydrides.

The optimized crystal structure of Na_2_LiXH_6_ (X = Zr, V, Cr) double perovskite hydrides demonstrates a cubic symmetry classified under the *Fm*3̄*m* (225) space group, as illustrated in Fig. 1. The atomic positions within the unit cell are specified as follows: Na atoms reside at the 8c (1/4, 3/4, 3/4) Wyckoff sites, Li atoms are located at 4b (1/2, 0, 0), X = (Zr/V/Cr) atoms reside at 4a (0, 0, 0), and H atoms are positioned at 24e (1/2, 0.718326, 0). The structural motif consists of alternating LiH_6_ and XH_6_ (X = Zr, V, Cr) octahedra, forming a three-dimensional framework wherein Na^+^ ions occupy the cuboctahedral cavities, consistent with the characteristic rock-salt arrangement of perovskite-type hydrides.^39^

Table 1 shows the optimized lattice parameters (*a* = *b* = *c*) and unit cell volumes (V) of Na_2_LiXH_6_ (X = Zr, V, Cr) double perovskite hydrides. The results reveal a gradual contraction in lattice dimensions, decreasing from 7.93 Å (Zr) to 7.33 Å (Cr). This gradual reduction in lattice dimension results from the decreasing ionic radii of the X-site cations, which enhances the crystal compactness and strengthens the metal–hydrogen interactions. The bonding environment serves a crucial role in function in controlling hydrogen retention and desorption behavior, where an optimal balance of bond strength facilitates hydrogen release at moderately low temperatures, beneficial to efficient storage-release cycles. In this context, the Goldschmidt tolerance factor (*τ*_G_), calculated using eqn (1),^40^ exhibits a slight increase from 0.88 for Na_2_LiZrH_6_ to 0.93 for Na_2_LiCrH_6_, remaining well within the stability range (0.8–1.0) characteristic of cubic double perovskite frameworks. This gradual rise in *τ*_G_ reflects enhanced structural symmetry and lattice compatibility, which contribute to the observed thermodynamic stability and favorable hydrogen desorption characteristics of the Cr-based compound. All compounds studied have octahedral values (*µ*) within the optimal range (0.42–0.75). These values confirm that all three compounds possess geometrically stable frameworks without significant distortion.1
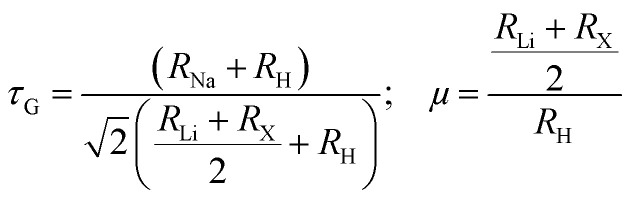
2
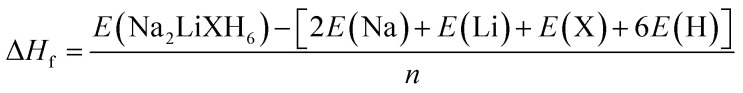
3
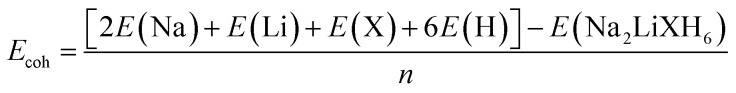


**Table 1:** Calculated structural parameters of Na_2_LiXH_6_ (X = Zr, V, Cr) perovskite hydrides

Compound	Lattice constant *a* = *b* = *c* (Å)	Volume (Å)^3^	*τ*_G_	*µ*	Δ*H*_f_ (eV per atom)	*E*_coh_ (eV per atom)
Na_2_LiZrH_6_	7.93	499.35	0.88	0.53	−1.26	+1.26
Na_2_LiVH_6_	7.45	414.38	0.92	0.46	−1.47	+1.47
Na_2_LiCrH_6_	7.33	393.57	0.93	0.45	−1.61	+1.61

The enthalpy of formation (Δ*H*_f_) further provides insight into the thermodynamic stability of the hydrides, which can be calculated using eqn (2).^41^ The calculated Δ*H*_f_ values of −1.26, −1.47, and −1.61 eV per atom for Na_2_LiZrH_6_, Na_2_LiVH_6_, and Na_2_LiCrH_6_, respectively, indicate an increasing exothermic trend, and *n* represents the total number of atoms. The progressively more negative formation enthalpies imply that substitution of Zr with V and Cr enhances the overall thermodynamic stability of the lattice. Although this trend corresponds with the decrease in the effective ionic size of the X-site cation, it is important to emphasise that the enhanced stability cannot be solely attributed to ionic radii. Instead, the observed exothermic behaviour is primarily governed by changes in electronic structure, bonding characteristics, and metal-hydrogen hybridization induced by the transition-metal substitution. Likewise, the cohesive energy (*E*_coh_) follows a similar sequence, increasing from +1.26 eV (Zr) to +1.61 eV (Cr), and was calculated using eqn (3),^42^ confirming stronger interatomic bonding as the X-site cation changes from Zr to Cr. The increase in cohesive strength is indicative of an augmentation in metal-hydrogen bonding interactions and an improvement in charge transfer. Rather than being a simple monotonic dependence on ionic radius alone, this phenomenon leads to enhanced lattice rigidity and a reduction in susceptibility to structural deformation. The combined structural, geometric, and energetic indicators reveal a consistent trend in the stability of the studied hydrides. Among the three, Na_2_LiCrH_6_ exhibits the highest structural and thermodynamic stability, followed by Na_2_LiVH_6_ and Na_2_LiZrH_6_. The enhanced compactness, favorable *τ*_G_ and higher *E*_coh_ collectively confirm the superior integrity of Na_2_LiCrH_6_ within the Na_2_LiXH_6_ series.

In addition to lattice parameters and formation enthalpies, the calculated M–H bond lengths provide significant insight into the bonding characteristics and hydrogen storage behavior of Na_2_LiXH_6_ (X = Zr, V, Cr) perovskite hydrides. The optimized structures exhibit short X–H bond lengths of 1.99 Å for Zr–H, 1.77 Å for V–H, and 1.71 Å for Cr–H, indicating strong M–H interactions within the [XH_6_] octahedra. The continuing contraction of the X–H bond from Zr to Cr reflects increasing orbital overlap between transition-metal d-states and H-state, consistent with enhanced covalent character and improved framework stability. In contrast, the significantly longer Na–H separations of 2.81 Å in Na_2_LiZrH_6_, 2.64 Å in Na_2_LiVH_6_, and 2.59 Å in Na_2_LiCrH_6_ show weaker ionic interactions between hydrogen and the A-site cations. This pronounced bonding asymmetry suggests that while H atoms coordinated to the X-site ensure structural rigidity, those associated with the A site are more-weakly bound and thus more amenable to thermally activated release. Such coexistence of strong X–H bonding and weaker A–H interactions is a defining structural feature governing hydrogen uptake and release kinetics in perovskite hydrides and has been widely recognized as a key factor in optimizing reversible solid-state H_2_ storage performance.^43,44^

### Dynamic stability

**Fig. 2:**
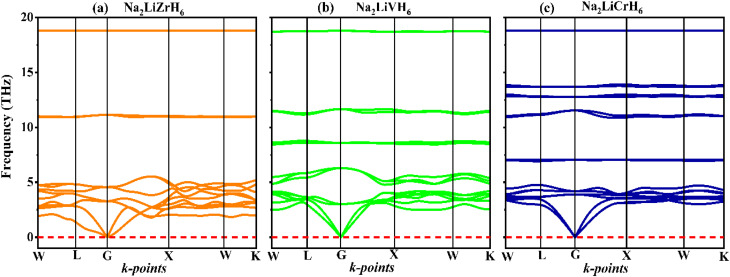
Phonon dispersions describing (a) Na_2_LiZrH_6_, (b) Na_2_LiVH_6_ and (c) Na_2_LiCrH_6_ perovskite hydrides.

Dynamic stability is a key factor governing the structural integrity and long-term functionality of materials, particularly in applications such as hydrogen storage, where materials must endure repeated thermal and mechanical cycles without degradation.^45^ To assess the dynamic stability of Na_2_LiXH_6_ (X = Zr, V, Cr) perovskite hydrides, phonon dispersion analysis was performed across the first Brillouin zone, as illustrated in Fig. 2a–c. Phonon dispersion relations serve as a fundamental tool for assessing the vibrational stability of crystalline materials, as they reveal how atomic vibrations propagate through the lattice. The appearance of imaginary (negative) phonon frequencies signifies dynamical instability, suggesting possible lattice distortions or phase transitions, whereas their absence confirms a stable crystal configuration suitable for practical applications. The lack of imaginary modes in the computed phonon band structures indicates that these compounds are dynamically stable under ambient conditions, ensuring their potential for practical applications.^46,47^

As depicted in Fig. 2a–c, Na_2_LiZrH_6_, Na_2_LiVH_6_, and Na_2_LiCrH_6_ all exhibit stable phonon dispersions, with no imaginary phonon modes observed across the Brillouin zone. The acoustic branches smoothly converge to zero near the Γ-point, whereas the optical modes remain clearly divided, displaying well-defined oscillatory mode features that are indicative of a structurally stable lattice.^48^ While the phonon frequencies vary slightly among the compounds, with Na_2_LiCrH_6_ showing higher frequencies indicative of a stiffer lattice, all three compounds maintain stable vibration profiles, suggesting that they are not susceptible to lattice distortions. The higher frequencies observed in Na_2_LiCrH_6_ indicate a stronger interatomic bond strength and more robust vibrational modes, potentially due to the higher atomic number and stronger bonding of Cr compared to Zr and V. This analysis confirms that the optimized structures of Na_2_LiZrH_6_, Na_2_LiVH_6_, and Na_2_LiCrH_6_ correspond to stable, minimum-energy states rather than saddle points, further supporting their dynamical stability and suitability for applications where long-term material performance is critical. The origin of the differences in phonon frequencies can be attributed to the differing electronic structures and bond strengths between the transition metals Zr, V, and Cr. The stronger metallic bonds in Na_2_LiCrH_6_ lead to higher phonon frequencies, reflecting a more rigid and robust lattice structure, while Na_2_LiZrH_6_ and Na_2_LiVH_6_ exhibit lower frequencies due to the weaker bonds in comparison. This gives rise to the subtle variation in dynamic behavior across these compounds.

The lack of low-frequency vibrational instabilities further verifies the vibrational robustness of these materials, indicating their ability to endure mechanical stresses while maintaining structural integrity. Additionally, the consistent stability observed across all three compounds reinforces their potential as reliable materials for high-temperature applications, such as H_2_ storage and clean energy technologies.

### *Ab initio* molecular dynamics (AIMD) calculations

**Fig. 3:**
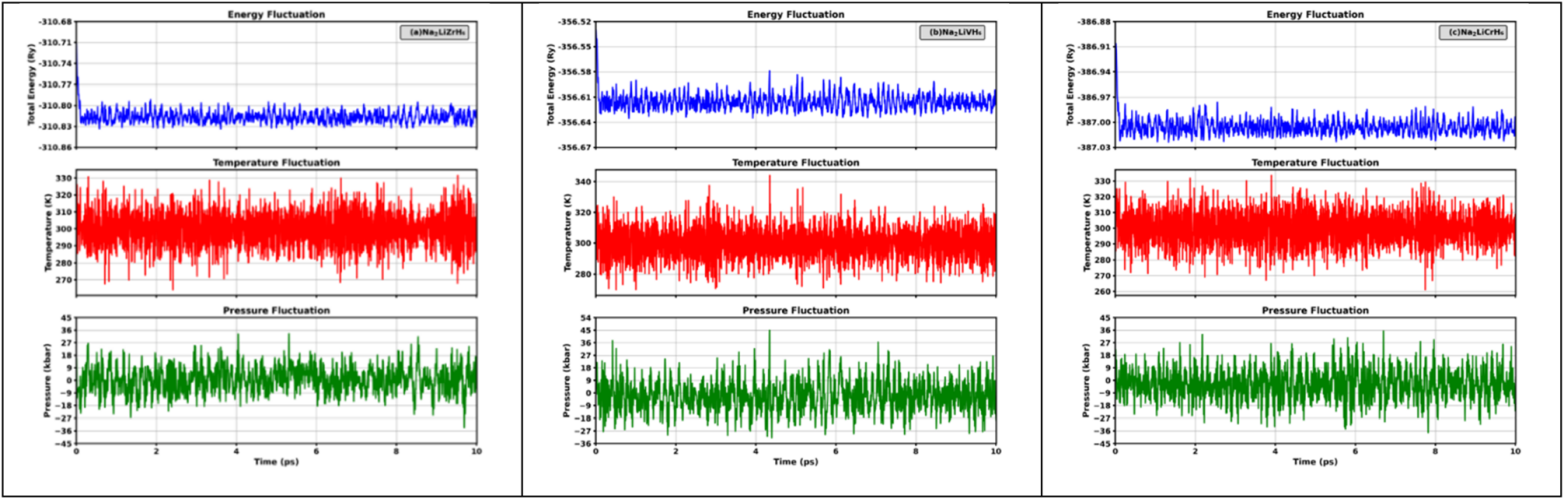
AIMD total-energy traces *versus* time for (a) Na_2_LiZrH_6_, (b) Na_2_LiVH_6,_ and (c) Na_2_LiCrH_6_ perovskite hydrides.

The thermal and dynamic stability of Na_2_LiXH_6_ (X = Zr, V, Cr) perovskite hydrides were systematically evaluated through AIMD simulations conducted at room temperature, as depicted in Fig. 3a–c. The results reveal strikingly similar behaviors across all three compounds, highlighting their robust thermodynamic stability. Na_2_LiZrH_6_, Na_2_LiVH_6_, and Na_2_LiCrH_6_ exhibit stable total energy profiles, with fluctuations confined within tightly constrained ranges, underscoring a lack of significant lattice degradation or phase transitions throughout the simulation period. Specifically, Na_2_LiZrH_6_ fluctuates between −310.86 and −310.71 Ry, Na_2_LiVH_6_ between −356.67 and −356.52 Ry, and Na_2_LiCrH_6_ between −387.03 and −386.91 Ry. These confined energy oscillations suggest that the systems are thermally stable and do not undergo any energetic instabilities, ensuring that their structural integrity remains intact under thermal perturbations. The slight difference in energy profiles across the compounds can be attributed to the differing electronic states of the transition metals. The higher binding energies in Na_2_LiCrH_6_ are likely due to the stronger bonding associated with Cr, contributing to its higher thermodynamic stability. The temperature fluctuations, oscillating around the equilibrium values of 300 K for all compounds, further confirm the sustained thermodynamic equilibrium throughout the simulations, with temperatures fluctuating between 270 and 330 K for Na_2_LiZrH_6_, 280 and 340 K for Na_2_LiVH_6_, and 270 and 320 K for Na_2_LiCrH_6_. These results underscore the durability of Na_2_LiXH_6_ compounds under realistic, dynamic conditions, which is crucial for their potential applications in energy storage.

In addition to energy and temperature stability, the pressure fluctuations observed across all three compounds remain periodic and within expected bounds, further demonstrating their structural resilience.^49^ The absence of any abrupt changes in pressure supports the notion that these perovskite hydrides can maintain their crystallographic framework under varying thermal and pressure conditions. Such stability is particularly important for materials intended for high-temperature applications, where they must endure fluctuating conditions without undergoing degradation or structural failure.^38,50^ These results are of significant importance for the potential application of Na_2_LiXH_6_ hydrides in energy storage systems, such as H_2_ storage, where materials must endure fluctuating temperatures and pressures without compromising their structural integrity. The differences in the pressure fluctuation behavior are minimal across the three compounds, with slight differences indicating the varying degrees of resistance to mechanical stress due to the electronic properties of Zr, V, and Cr. The consistent stability observed across Na_2_LiZrH_6_, Na_2_LiVH_6_, and Na_2_LiCrH_6_ positions these compounds as promising candidates for use in demanding high-temperature applications, reinforcing their potential for advancing clean energy technologies.

### Hydrogen storage

Hydrogen is a dynamic, sustainable energy carrier with an unparalleled gravimetric energy density and flawlessly clean combustion, making it an essential part of the shift to carbon-neutral systems as the world looks for the next generation of clean power. However, a significant obstacle to its broad use is still the absence of safe, effective, and compact storage solutions. In this context, complex hydrides, particularly double perovskite-type hydrides, have attracted increasing theoretical interest due to their high hydrogen content and structural tunability. However, Na_2_LiXH_6_ (X = Zr, V, Cr) perovskite hydrides remain largely unexplored in the literature, especially with respect to a systematic first-principles assessment of their stability and hydrogen-related properties.

Based on our first-principles calculations, Na_2_LiXH_6_ (X = Zr, V, Cr) exhibit favorable structural and dynamical stability, which motivates their consideration as potential candidates for solid-state hydrogen storage rather than established storage materials. As seen in Table 2, the H_2_ storage capacity of Na_2_LiXH_6_ (X = Zr, V, Cr) was evaluated in terms of gravimetric capacity (*C*_wt%_), volumetric capacity (*ρ*_vol_), and desorption temperature (*T*_des_). Eqn (4) was used to calculate the gravimetric H_2_ capacity, which is defined as the ratio of hydrogen mass to the total mass of the hydride.^51^4
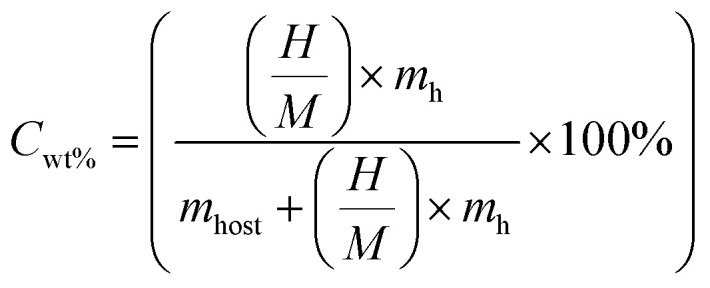
where *m*_h_ and *m*_host_ represent the molar masses of hydrogen and the host material, respectively. Na_2_LiZrH_6_, Na_2_LiVH_6_, and Na_2_LiCrH_6_ have determined *C*_wt%_ of 4.03 weight percent, 5.50 weight percent, and 5.45 weight percent, respectively. Among these, Na_2_LiVH_6_ and Na_2_LiCrH_6_ show potential as high-capacity H_2_ storage materials by getting close to the U.S. DOE 2025 objective of 5.5 weight percent. The decrease in molecular weight and increased hydrogen bonding strength within the lattice, which increases hydrogen uptake efficiency, are the main causes of the rise in *C*_wt%_ from Zr to V and Cr replacement. Another crucial performance parameter for real-world storage applications where compactness is necessary is the volumetric H_2_ capacity (*ρ*_vol_), which shows the hydrogen content per unit volume. It was evaluated using eqn (5).^52^5
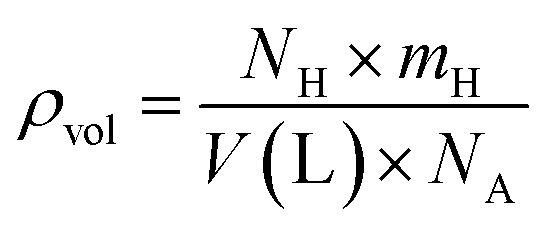


**Table 2:** The *ρ*_vol_, *T*_des_ and gravimetric ratios of Na_2_LiXH_6_ (X = Zr, V, Cr) perovskite hydrides

Compound	*C*_wt_ (%)	Δ*H*_f_ (kJ per mol per H_2_)	*ρ*_vol_ (g per H_2_ per L)	*T*_des_ (K)	References
K_2_NaAlH_6_	4.47	—	74.25	484.52	56
Cs_2_NaInH_6_	1.48	—	14.18	492.70	57
K_2_LiTiH_6_	4.38	−56.96	19.12	435.80	46
Ca_2_LiCuH_6_	3.86	—	15.68	717.20	39
Na_2_LiZrH_6_	4.03	−60.54	20.11	463.16	Present study
Na_2_LiVH_6_	5.50	−70.61	24.24	540.23	Present study
Na_2_LiCrH_6_	5.45	−77.69	25.52	594.44	Present study

The *ρ*_vol_ were determined to be 20.11 g per H_2_ per L for Na_2_LiZrH_6_, 24.24 g per H_2_ per L for Na_2_LiVH_6_, and 25.52 g per H_2_ per L for Na_2_LiCrH_6_, as indicated in Table 2. Denser atomic packing and stronger lattice cohesion increase hydrogen density per unit volume, as indicated by the increasing trend in *ρ*_vol_ from Zr to Cr replacement. This is beneficial for applications requiring both high energy density and compact storage. When evaluating a storage material's viability, especially for fuel cell integration, hydrogen desorption is represented by the desorption temperature (*T*_des_) is crucial. Gibbs free energy governs the desorption process thermodynamically and is characterized by (eqn 6) and (7).^53,54^

6 Δ*G* = Δ*H* − *T*_des_ × Δ*S*

7
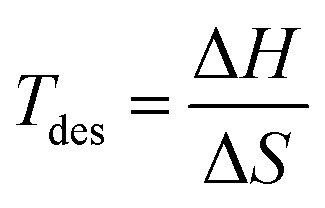
where the enthalpy and entropy changes during desorption are represented by Δ*H* and Δ*S*. According to the computed results, *T*_des_ of Na_2_LiZrH_6_, Na_2_LiVH_6_, and Na_2_LiCrH_6_ are 463.16, 540.23, and 594.44 K, respectively. Stronger metal-hydrogen interactions inside the lattice are correlated with a progressive increase in *T*_des_, especially for Cr-substituted systems. These temperatures show that these materials are thermodynamically stable and capable of reversible hydrogen release under moderate heating conditions, even if they are somewhat higher than the optimal range for direct PEM fuel cell operation (233–333 K).^55^ In brief, both gravimetric and volumetric measurements demonstrate that Na_2_LiVH_6_ and Na_2_LiCrH_6_ outperform Na_2_LiZrH_6_ in terms of H_2_ storage capacity and stability. The higher desorption enthalpy and temperature values for Na_2_LiCrH_6_ confirm stronger M–H bonding and improved thermal resistance, which makes it particularly attractive for high-temperature H_2_ storage and transport applications. The present results demonstrate that controlled transition-metal substitution can effectively tune the hydrogen-related properties of Na_2_LiXH_6_ perovskite hydrides. While the present analyses are based on thermodynamic stability, phonon dispersion, and AIMD simulations, they establish a theoretical foundation that motivates future experimental validation and kinetic studies.

### Mechanical properties

The mechanical properties serve as a crucial indicator of the structure-dependent elastic response in perovskite hydrides. Accurate determination of second-order elastic constants (*C*_ij_) is fundamental for evaluating mechanical stability and understanding a material's resistance to external deformation and applied stress. In the present study, the elastic constants of Na_2_LiXH_6_ (X = Zr, V, Cr) were systematically computed, from which essential mechanical moduli and anisotropic factors were derived to provide a comprehensive assessment of their stability and mechanical resilience. The obtained parameters not only validate the mechanical robustness of these compounds but further elucidate their deformability, rigidity, and bonding nature, which are key determinants of their reliability and efficiency under hydrogen storage conditions. The elastic response of Na_2_LiXH_6_ (X = Zr, V, Cr) was characterized by computing the distinct elastic coefficients *C*_11_, *C*_12_, and *C*_44_ corresponding to the cubic symmetry; the full set of derived moduli is reported in Table 3. The calculated *C*_11_, *C*_12_, and *C*_44_ values increase from Zr to Cr, indicating that substitution at the X site produces a progressive stiffening of the lattice. Physically, the rising *C*_11_ and *C*_44_ reflect enhanced resistance to longitudinal and shear deformations, respectively, as the X-site cation is varied from Zr to Cr.

**Table 3:** The computed elastic moduli of Na_2_LiXH_6_ (X = Zr, V, Cr) perovskite hydrides

Elastic moduli	Na_2_LiZrH_6_	Na_2_LiVH_6_	Na_2_LiCrH_6_
C_11_	47.74	71.24	93.23
*C*_12_	15.58	22.43	28.15
*C*_44_	18.45	26.74	30.15
*C*_p_	−2.87	−4.31	−2.0
*B*(GPa)	26.29	38.69	49.85
*G*(GPa)	17.46	25.78	31.08
*E*(GPa)	42.89	63.28	77.20
*B*/*G*	1.51	1.50	1.60
*υ*	0.22	0.23	0.24
*C*′	16.08	24.41	32.54
*A* ^U^	1.15	1.10	0.93

All three compounds satisfy the Born mechanical criteria for cubic symmetry, namely *C*_11_ − *C*_12_ > 0, *C*_11_ + 2*C*_12_ > 0, and *C*_44_ > 0. In particular, the shear constant (*C*′ = (*C*_11_ − *C*_12_)/2) is positive in every case (16.08, 24.41, and 32.54 GPa), confirming mechanical stability against shear modes and indicating that the relaxed structures are mechanically robust (Table 3).

The bulk modulus (*B*) increases progressively from 26.29 to 38.69 to 49.85 GPa, while the shear modulus (*G*) rises from 17.46 to 25.78 to 31.08 GPa, and the Young's modulus (*E*) increases from 42.89 to 63.28 to 77.20 GPa. All elastic moduli were computed using eqn (8)–(12).^58–61^ This consistent enhancement in elastic moduli demonstrates that Na_2_LiCrH_6_ exhibits the greatest stiffness and lowest compressibility within the series. The systematic improvement in elastic stiffness is closely associated with the observed reduction in lattice constants and the increase in cohesive energy, suggesting that the Cr-based compound possesses superior mechanical integrity under both hydrostatic and shear stresses.

**Fig. 4:**
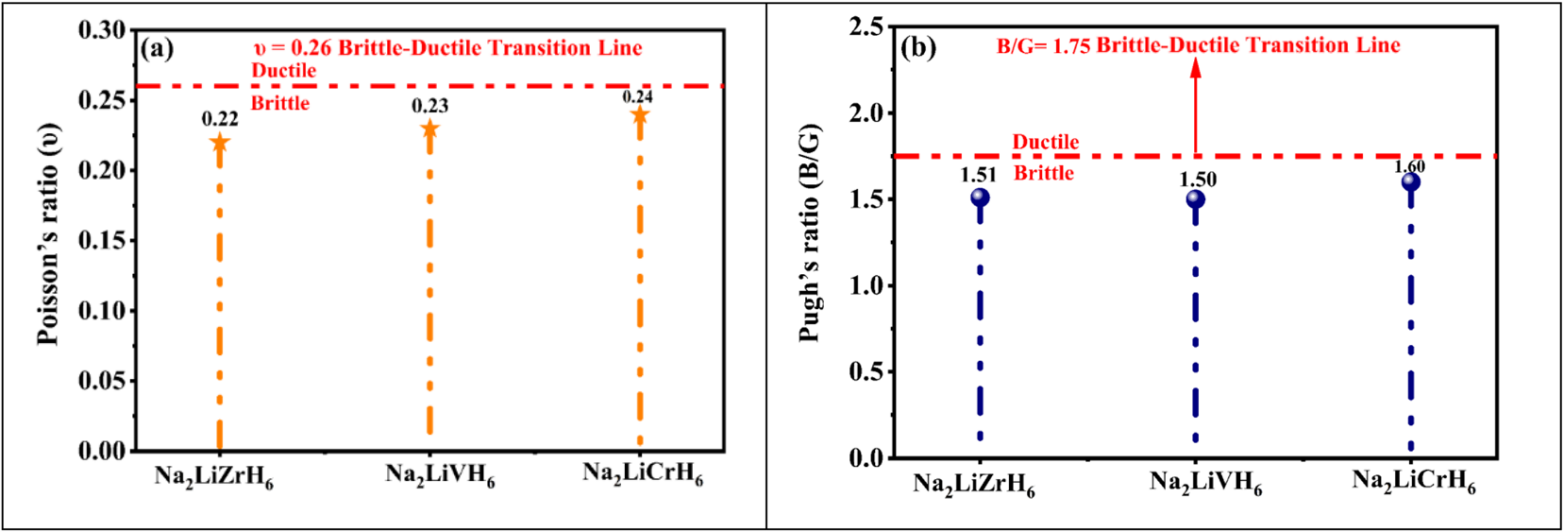
Graphs of (a) Poisson's ratio (*υ*), and (b) Pugh's ratio (*B*/*G*)of Na_2_LiXH_6_ (X = Zr, V, Cr) perovskite hydrides.

Ductility/brittleness was assessed using Pugh's ratio (*B*/*G*) and Poisson's ratio (*υ*) as shown in Fig. 4. The computed *B*/*G* values (1.51, 1.50, and 1.60) are all below the empirical ductile threshold of ≈1.75, and *υ* (0.22, 0.23, and 0.24) remain below 0.26; both criteria therefore classify these materials as predominantly brittle with limited plasticity. Nonetheless, the slight increase of *B*/*G* and *υ* for Na_2_LiCrH_6_ indicates a modest shift toward less brittle behavior relative to the Zr- and V-variants. Cauchy pressure, defined as *C*_p_ = *C*_12_ − *C*_44_, is negative for all three compositions (−2.87, −4.31, and −2.00 GPa). A negative *C*_p_ generally signals significant directional (covalent-like) bonding contributions; thus, the elastic data imply that the bonding in these hydrides has a notable non-ionic/covalent component, consistent with strong X–H and M–H interactions in the framework. Elastic anisotropy was quantified using the universal anisotropy index *A*^U^. The computed *A*^U^ values (1.15, 1.10, and 0.93) show that Na_2_LiCrH_6_ is the most nearly isotropic (*A*^U^ ≈ 1), whereas Na_2_LiZrH_6_ and Na_2_LiVH_6_ display modest anisotropy. The presence of finite anisotropy, particularly in the Zr and V compounds, suggests directional dependence of mechanical response that should be considered when designing single crystals, thin films, or polycrystalline components. The elastic data verify that all three Na_2_LiXH_6_ perovskites are mechanically stable in the cubic phase. Progressive substitution from Zr to V to Cr strengthens the lattice, as reflected by the higher values of *C*_*ij*_, *B*, *G*, and *E*, and slightly decreases brittleness. Among them, Na_2_LiCrH_6_ exhibits the most favorable combination of stiffness and nearly isotropic elastic behavior. These mechanical characteristics, enhanced rigidity and structural stability for the Cr-based compound, are advantageous for practical handling and integration into devices. However, the inherently brittle nature of these materials may necessitate microstructural engineering strategies, such as the development of composites or protective coatings, to enable their use in applications requiring greater mechanical flexibility. Such elastic attributes significantly reinforce the structural stability and adaptability of these compounds, thereby emphasizing their strong potential for efficient hydrogen (H_2_) storage applications.8
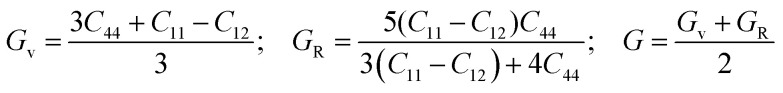
9
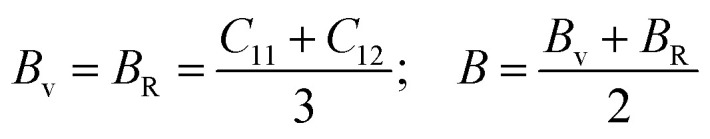
10
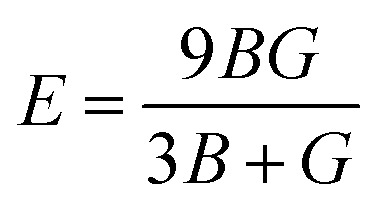
11
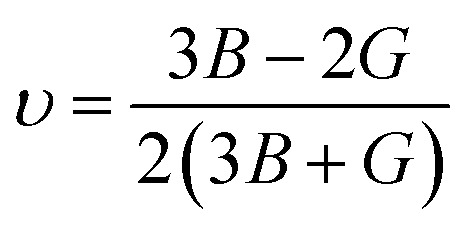
12
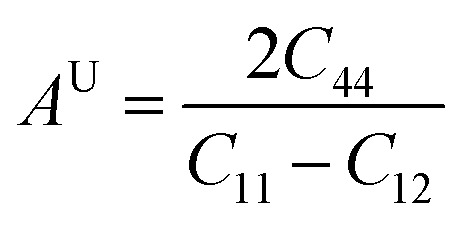


### Electronic properties

**Fig. 5:**
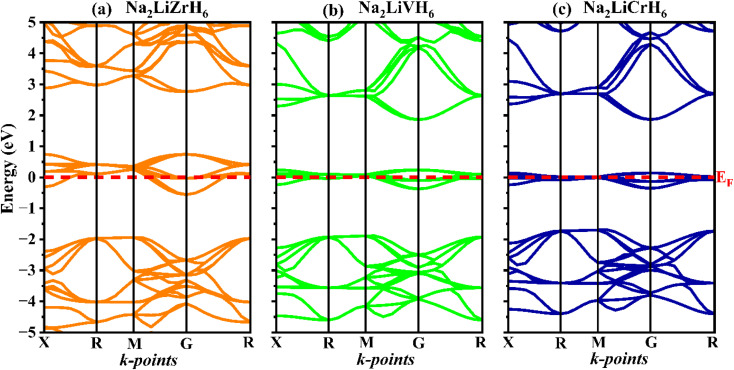
Computed band structures of describing (a) Na_2_LiZrH_6_, (b) Na_2_LiVH_6_ and (c) Na_2_LiCrH_6_ perovskite hydrides.

Electronic characteristics, especially the energy bandgap (*E*_g_) and density of states (DOS), serve a vital function in controlling the adsorption and desorption behavior, structure stability, as well as catalytic activity of hydrogen storage materials, thereby facilitating efficient hydrogen dissociation and recombination processes. A detailed understanding of the electronic band structure is fundamental to elucidating the electrical conductivity and optical behaviour of crystalline hydrides. It provides insights into energy distribution and bonding characteristics within the material. Fig. 5 presents the calculated band structures of Na_2_LiXH_6_ (X = Zr, V, Cr) along the high symmetry path X–R–M–Γ–R, obtained using the GGA-PBE functional. The Fermi level is indicated by a dashed olive line. All three compounds display an overlap at the Fermi level, with the valence band maximum (VBM) and conduction band minimum (CBM) located at distinct symmetry points, confirming the absence of a bandgap and indicating their metallic nature. The valence bands (VB) near the Fermi level appear relatively flat, suggesting localized bonding states, while the conduction bands (CB) show greater dispersion, implying enhanced electron mobility. With substitution from Zr to V to Cr, there is a gradual increase in the dispersion of the conduction bands and a narrowing of the valence bandwidth, reflecting subtle electronic restructuring across the series. These observations are key to understanding the DOS and optical response discussed in the subsequent section.

**Fig. 6:**
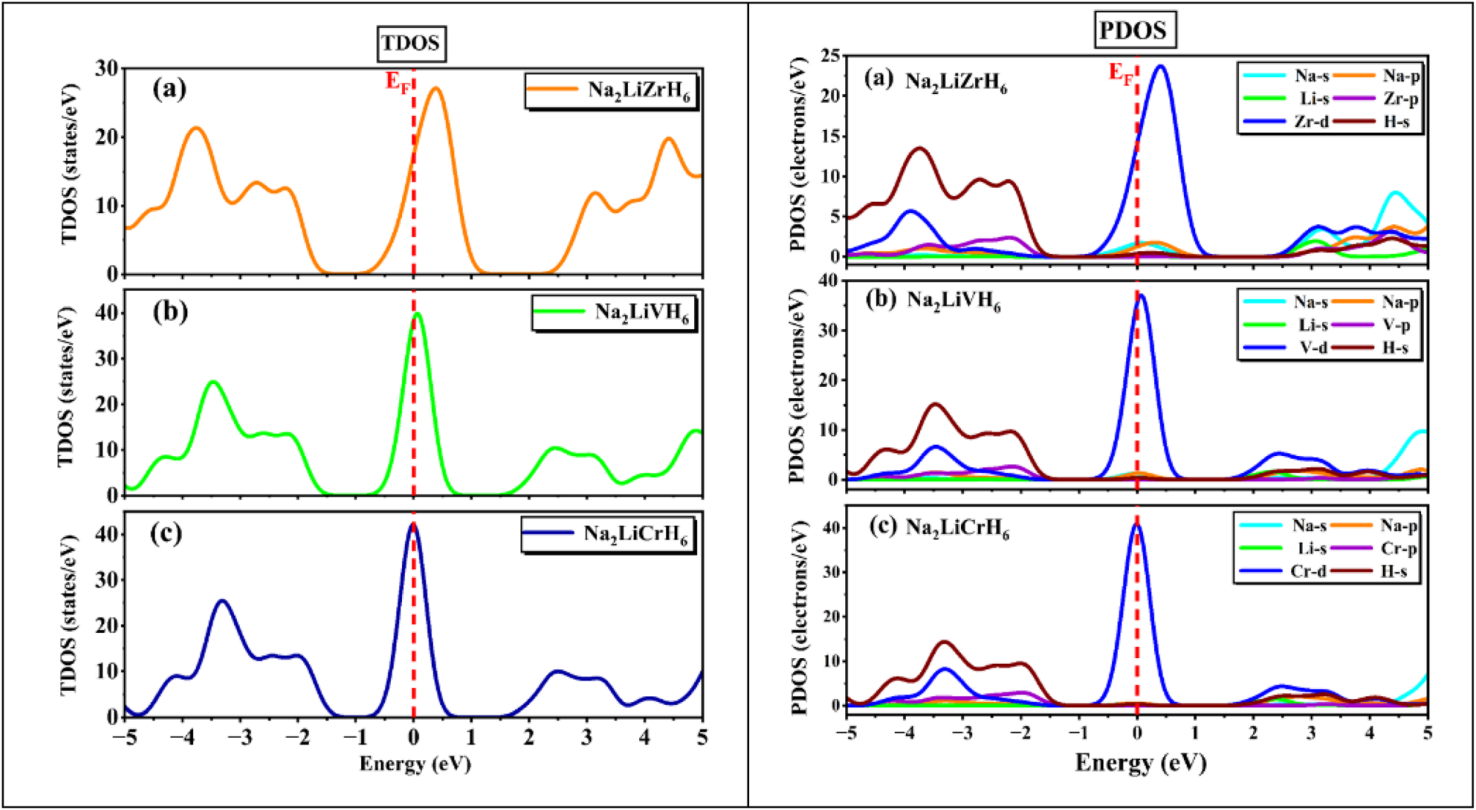
TDOS and PDOS for Na_2_LiXH_6_ (X = Zr, V, Cr) perovskite hydrides.

Fig. 6 presents the total and partial density of states (PDOS) for Na_2_LiXH_6_ (X = Zr, V, Cr) hydrides, offering comprehensive insights into their electronic characteristics and bonding nature. In all three systems, the Fermi level (*E*_F_) intersects prominent peaks, indicating metallic characteristics and significant electronic delocalization. For Na_2_LiZrH_6_, the VB region from about −5 eV up to the *E*_F_ is mainly formed by the hybridized Zr-d and H-s orbitals, with minor contributions from Na-s and Li-s states. The CB is primarily governed by Zr-d states, indicating pronounced metal-hydrogen interactions near the *E*_F_. In the case of Na_2_LiVH_6_, the top of the VB primarily originates from V-d and H-s orbitals, while the CB reveals further hybridization between V-d and H-s states, pointing to strong covalent interactions within the VH_6_ octahedra. Likewise, Na_2_LiCrH_6_ exhibits intense mixing between Cr-d and H-s orbitals near the *E*_F_, where the partially filled Cr-d states play a significant role in verifying the electronic character. This pronounced d-s hybridization suggests a comparatively more covalent Cr–H bonding character than in the Zr- and V-based counterparts.

As the transition metal changes from Zr to V and then to Cr, the increasing covalent contribution reduces the purely ionic stabilization of H^−^, which may facilitate hydrogen mobility and lower the dehydrogenation barrier. Consequently, while Na_2_LiCrH_6_ exhibits strong metal–hydrogen interactions and high thermal stability, its hydrogen storage behavior should be viewed as a balance between enhanced bonding strength and potentially improved hydrogen release kinetics rather than exclusively superior high-temperature storage performance. Overall, the PDOS analysis confirms that Na_2_LiXH_6_ (X = Zr, V, Cr) exhibits metallic behavior at the *E*_F_, enabling rapid charge transport and promoting efficient hydrogen storage-release dynamics.

### Optical properties

**Fig. 7:**
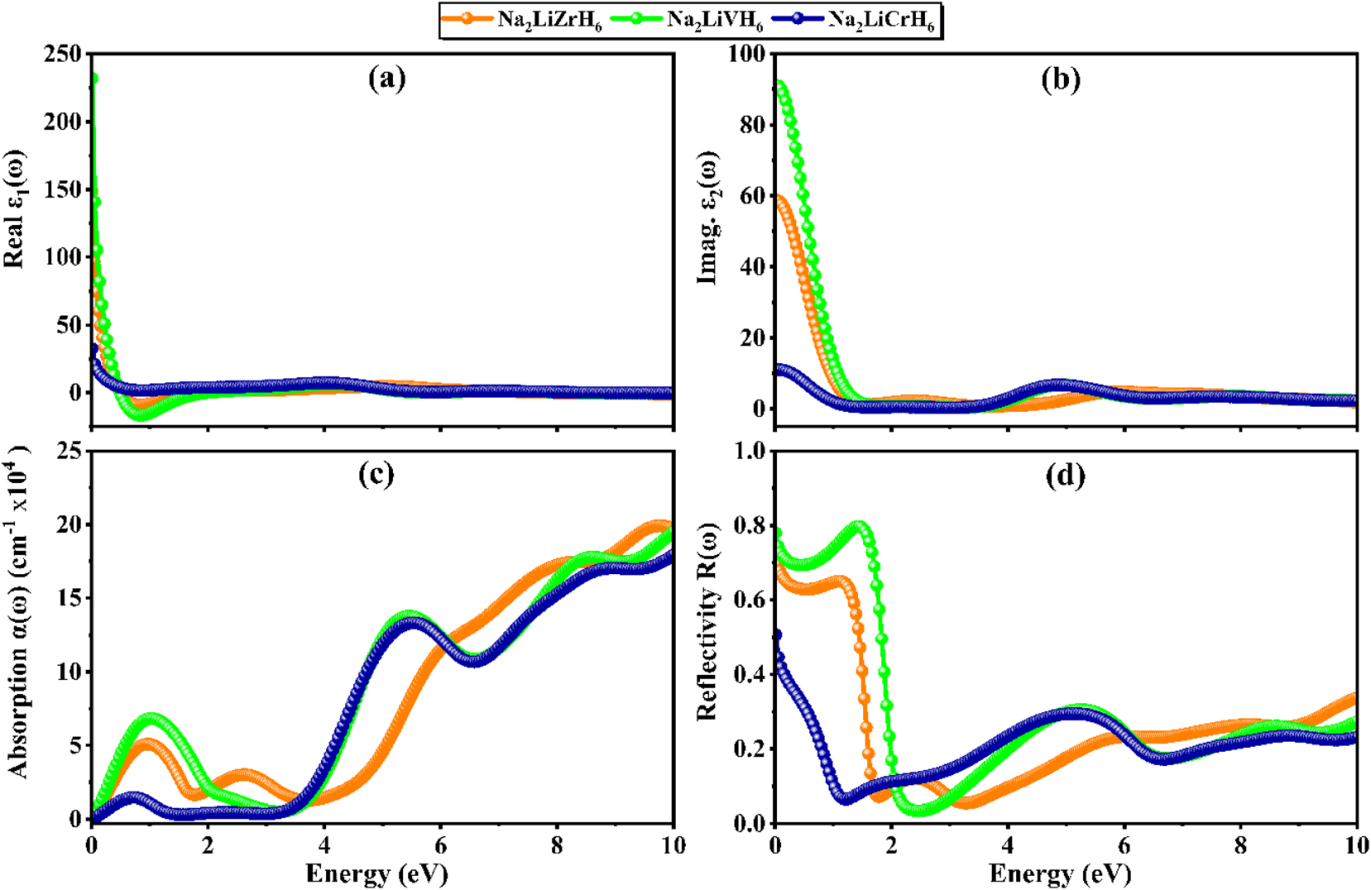
Optical response of Na_2_LiXH_6_ (X = Zr, V, Cr) perovskite hydrides.

Optical properties offer valuable information on the interaction between materials and incident electromagnetic waves, revealing the bandgap nature, electronic transition mechanisms, and hydrogen adsorption and desorption behavior. These attributes are fundamental for assessing the potential of hydride-based compounds in advanced functional and energy-related applications. In the context of hydrogen storage, the metallic or insulating nature of a material critically influences thermal and electronic transport during hydrogen uptake and release.^62–65^ Metallic behavior, associated with enhanced electronic and thermal conductivity, can promote efficient heat management and improved cycling kinetics, whereas insulating or wide-bandgap characteristics may reduce electronic losses and contribute to structural stability.^66^ The optical response discussed herein is fully consistent with this electronic classification, where metallic systems exhibit pronounced low-energy absorption without a distinct optical band edge, while insulating or semiconducting compounds show a clear absorption onset arising from interband transitions. This correlation establishes a coherent link between optical behavior, electronic structure, and hydrogen storage performance. Fig. 7 shows the computed optical properties of Na_2_LiXH_6_ (X = Zr, V, Cr) in the photon energy range 0–10 eV, including the real [*ε*_1_(*ω*)] and imaginary [*ε*_2_(*ω*)] parts of the dielectric function, absorption coefficient [*α*(*ω*)], and reflectivity *R*(*ω*). The trends in these spectra are consistent with the previously discussed electronic structures, highlighting the influence of the transition-metal cation on light–matter interaction. Fig. 7a illustrates the real part of the dielectric function, *ε*_1_(*ω*), where Na_2_LiVH_6_ displays the highest static dielectric constant, signifying stronger polarization and enhanced electronic screening. Na_2_LiZrH_6_ exhibits a comparatively moderate response, whereas Na_2_LiCrH_6_ shows the lowest value, indicating reduced polarizability. Hence, the pronounced dielectric response of Na_2_LiVH_6_ enhances its suitability for hydrogen storage systems, as efficient polarization promotes effective charge transfer and strengthens the overall adsorption–desorption dynamics. The imaginary part *ε*_2_(*ω*) (Fig. 7b) demonstrates a similar order, where Na_2_LiVH_6_ and Na_2_LiZrH_6_ possess pronounced low-energy peaks arising from interband transitions near the Fermi level, whereas Na_2_LiCrH_6_ displays weaker intensity and a shift of major optical activity toward higher energies. Hence, the distinct interband transitions observed in Na_2_LiCrH_6_ enhanced its potential for hydrogen storage applications by promoting light-induced electronic excitation and facilitating efficient hydrogen desorption processes.

The absorption spectra (Fig. 7c) confirm that Na_2_LiVH_6_ and Na_2_LiZrH_6_ absorb significantly in the visible and near-UV regions, with multiple absorption edges starting near their band gaps, whereas Na_2_LiCrH_6_ exhibits a sharp increase only in the ultraviolet domain, consistent with its wider gap. Reflectivity (Fig. 7d) trends complement this observation: Na_2_LiVH_6_ and Na_2_LiZrH_6_ exhibit high low-energy reflectance (>0.5), indicating reduced transmission, whereas Na_2_LiCrH_6_ maintains relatively low reflectivity and a smoother spectral variation. Hence, Na_2_LiVH_6_ and Na_2_LiZrH_6_ demonstrate strong optical screening and intense interband transitions, making them promising for broadband absorption-based devices, whereas Na_2_LiCrH_6_, with its moderate dielectric response and dominant UV absorption, stands out as a potential candidate for ultraviolet optoelectronic or protective coating applications.

### Thermal properties

The thermodynamic properties of Na_2_LiXH_6_ (X = Zr, V, Cr) perovskite hydrides are crucial in assessing their capabilities for hydrogen storage and release mechanisms. Favorable thermodynamic characteristics in H_2_ storage materials facilitate effective cycles of hydrogen absorption and desorption, which are essential for preserving reversibility and long-term stability. The structural response of these hydrides to temperature changes and their capacity to efficiently store and release hydrogen can be better understood by examining characteristics like heat capacity, enthalpy, entropy, and free energy. If the hydrides' thermodynamic profile remains constant, they can endure multiple cycles of hydrogenation and dehydrogenation without experiencing noticeable degradation in structure.^67^ Examining thermodynamic parameters like longitudinal sound velocity (*v*_l_), transverse sound velocity (*v*_t_), average sound velocity (*v*_m_), Debye temperature (*θ*_D_), and melting temperature (*T*_m_) is essential for understanding the vibrational and lattice dynamics that affect hydrogen diffusion and lattice resilience at high temperatures. Eqn (13)–(15) were used to derive these parameters:^68,69^13
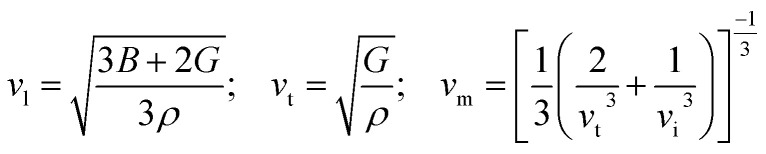
14
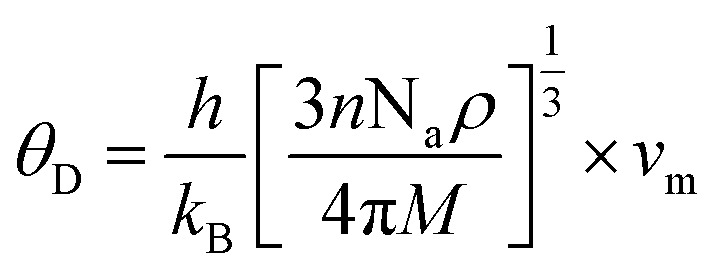

15 *T*_m_ = [553 + 5.911(*C*_11_)] ± 300

where *ρ* is the material density, *M* is the molecular weight, *C*_11_ is the elastic constant, and *B* and *G* are the bulk and shear moduli. These equations connect the material's thermal and dynamic stability, which is essential for H_2_ storage devices that operate in temperature-varying environments, to mechanical stiffness and atomic vibrations.

**Fig. 8:**
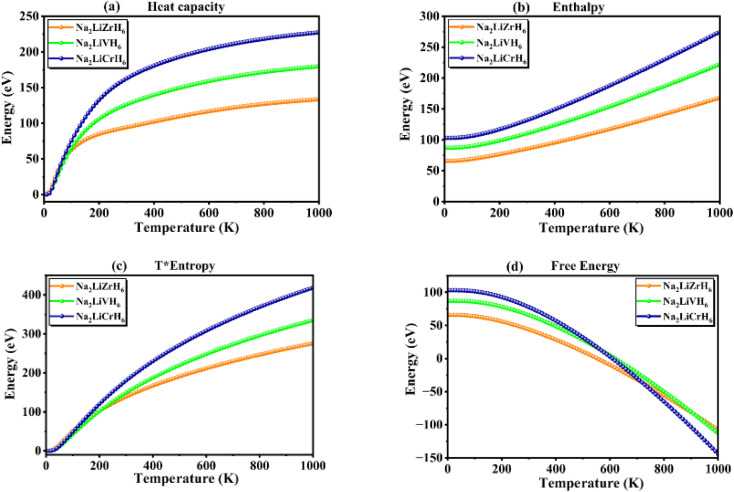
Thermal parameters of Na_2_LiXH_6_ (X = Zr, V, Cr) perovskite hydrides.

All three compounds (Na_2_LiZrH_6_, Na_2_LiVH_6_, and Na_2_LiCrH_6_) show identical thermodynamic trends with increasing temperature, as shown in Fig. 8a–d and detailed in Table 4. At increasing temperatures, the heat capacity (Fig. 8a) and enthalpy (Fig. 8b) increase monotonically, indicating increased phonon contributions and lattice vibration amplitudes. Notably, Na_2_LiCrH_6_ exhibits the highest enthalpy and heat capacity values, indicating improved energy storage and stronger interatomic bonding. The vibrational and configurational entropy contributions grow with temperature, supporting the desorption process by lowering the Gibbs free energy barrier, as the total entropy (Fig. 8c) rises linearly. Fig. 8d shows that all compounds exhibit a consistent decline in free energy as the temperature rises, a typical behavior of thermodynamically stable hydrides that facilitates hydrogen release at higher temperatures.

**Table 4:** Calculated thermodynamic parameters of Na_2_LiXH_6_ (X = Zr, V, Cr) perovskite hydrides

Parameters	Na_2_LiZrH_6_	Na_2_LiVH_6_	Na_2_LiCrH_6_
*v*_l_ (km s^−1^)	3.08	3.74	4.20
*v*_t_ (km s^−1^)	2.09	2.54	2.79
*v*_m_ (km s^−1^)	2.28	2.77	3.05
*θ*_D_ (K)	369	497	546
*T*_m_ (K)	835	974	1104

The most reliable thermodynamic performance is shown quantitatively by Na_2_LiCrH_6_ with *v*_l_ = 4.20 km s^−1^, *v*_t_ = 2.79 km s^−1^, *v*_m_ = 3.05 km s^−1^, *θ*_D_ = 546 K, and *T*_m_ = 1104 K, signifying higher lattice stiffness and strong atomic interactions. Na_2_LiZrH_6_ has slightly lower values (*θ*_D_ = 369 K, *T*_m_ = 835 K), suggesting a relatively softer lattice, while Na_2_LiVH_6_ follows closely (*θ*_D_ = 497 K, *T*_m_ = 974 K). By increasing vibrational frequency and phonon transport efficiency, Cr inclusion strengthens lattice rigidity and improves thermal stability, as demonstrated by the observed rise in Debye temperature from Zr to Cr substitution. Na_2_LiCrH_6_'s mechanical and thermal endurance during hydrogen cycling is improved by its higher Debye temperature, which indicates a higher phonon frequency and a smaller atomic vibration amplitude. These findings demonstrate that Na_2_LiCrH_6_ has the strongest lattice among the compounds under investigation, which makes it a viable option for H_2_ storage applications where effective absorption–desorption performance depends on high thermal and structural stability.

## Conclusion

In summary, this in-depth first-principles investigation elucidates the intrinsic correlation between the structural, mechanical, electronic, optical, and thermal characteristics of Na_2_LiXH_6_ (X = Zr, V, Cr) double perovskite hydrides. The compounds crystallize to form a cubic *Fm*3̄*m* structure, showing negative formation energies and favourable tolerance factors. These factors indicate that these compounds are structurally and thermodynamically stable. All compounds maintain mechanical and thermodynamic stability within the cubic phase, exhibiting progressive improvements in elastic stiffness, cohesive strength, and lattice integrity from Zr to Cr substitution. Among the studied systems, Na_2_LiVH_6_ demonstrates the most resilient configuration, supported by its bulk modulus, Debye temperature (597 K), melting point (974 K), and sound velocities, confirming strong atomic bonding and high thermal endurance. Its favorable desorption enthalpy (−70.61 kJ per mol per H_2_) and volumetric hydrogen density (24.24 g per H_2_ per L) further verify its superior hydrogen storage capability. In addition, the compound's broad optical absorption and pronounced dielectric response suggest potential for dual hydrogen-optoelectronic functionality. Collectively, the synergistic combination of mechanical rigidity, thermal stability, and optical activity designates Na_2_LiVH_6_ as a potential multifunctional material for sustainable hydrogen storage and advanced optoelectronic technologies.

## Ethical statement

Approval all authors affirm that this submission is entirely original, has not been published elsewhere, and fully adheres to the established ethical standards for scholarly research and publication.

## Author contributions

All authors affirm their substantial contributions to this research and collectively assume full responsibility for its content, encompassing conceptualization, computational analysis, manuscript preparation, and revision processes.

## Conflicts of interest

The authors declare that there are no financial or personal relationships that could have influenced the outcomes, interpretation, or conclusions presented in this study.

## Data Availability

The data will be provided on request by the corresponding author. Supplementary information (SI) is available. See DOI: https://doi.org/10.1039/d5ra08708b.
